# The Somatostatin Receptor-4 Agonist J-2156 Alleviates Mechanical Hypersensitivity in a Rat Model of Breast Cancer Induced Bone Pain

**DOI:** 10.3389/fphar.2018.00495

**Published:** 2018-05-15

**Authors:** Priyank A. Shenoy, Andy Kuo, Nemat Khan, Louise Gorham, Janet R. Nicholson, Laura Corradini, Irina Vetter, Maree T. Smith

**Affiliations:** ^1^Faculty of Medicine, School of Biomedical Sciences, The University of Queensland, Brisbane, QLD, Australia; ^2^Department of CNS Diseases Research, Boehringer Ingelheim Pharma GmbH & Co. KG, Biberach, Germany; ^3^Institute for Molecular Bioscience, The University of Queensland, Brisbane, QLD, Australia; ^4^Faculty of Health and Behavioural Sciences, School of Pharmacy, The University of Queensland, Brisbane, QLD, Australia

**Keywords:** J-2156, somatostatin receptor 4 agonist, mechanical hypersensitivity, mechanical allodynia, mechanical hyperalgesia, Walker 256 cells, rat model of breast cancer induced bone pain

## Abstract

In the majority of patients with breast cancer in the advanced stages, skeletal metastases are common, which may cause excruciating pain. Currently available drug treatments for relief of breast cancer-induced bone pain (BCIBP) include non-steroidal anti-inflammatory drugs and strong opioid analgesics along with inhibitors of osteoclast activity such as bisphosphonates and monoclonal antibodies such as denosumab. However, these medications often lack efficacy and/or they may produce serious dose-limiting side effects. In the present study, we show that J-2156, a somatostatin receptor type 4 (SST4 receptor) selective agonist, reverses pain-like behaviors in a rat model of BCIBP induced by unilateral intra-tibial injection of Walker 256 breast cancer cells. Following intraperitoneal administration, the ED_50_ of J-2156 for the relief of mechanical allodynia and mechanical hyperalgesia in the ipsilateral hindpaws was 3.7 and 8.0 mg/kg, respectively. Importantly, the vast majority of somatosensory neurons in the dorsal root ganglia including small diameter C-fibers and medium-large diameter fibers, that play a crucial role in cancer pain hypersensitivities, expressed the SST4 receptor. J-2156 mediated pain relief in BCIBP-rats was confirmed by observations of a reduction in the levels of phosphorylated extracellular signal-regulated kinase (pERK), a protein essential for central sensitization and persistent pain, in the spinal dorsal horn. Our results demonstrate the potential of the SST4 receptor as a pharmacological target for relief of BCIBP and we anticipate the present work to be a starting point for further mechanism-based studies.

## Introduction

Breast cancer is the most frequent type of cancer diagnosed in women and the major cause of cancer-associated mortalities in the world (DeSantis et al., 2015). Metastasis of breast cancer cells to the skeleton is a significant problem as it may cause excruciating pain (Bu et al., 2014). Bone metastases lead to destruction of bones due to increased activity of osteoclasts (Kane et al., 2015). Cancer cells in the bones locally stimulate as well as induce the release of inflammatory mediators (Lozano-Ondoua et al., 2013; Esquivel-Velázquez et al., 2015; Kane et al., 2015). Sensory nerve fibers innervating tumor bearing bones, undergo pathological sprouting and reorganization (Bloom et al., 2011). Hence, cancer-induced bone pain has a very complex pathophysiology as it is underpinned by both inflammatory and neuropathic components, along with an interplay of cancer-specific factors (Cao et al., 2010). It involves pathobiological alterations of peripheral tissues and nerve fibers as well as characteristic neurochemical changes at the level of the spinal cord (Falk and Dickenson, 2014).

Presently, non-steroidal anti-inflammatory drugs (NSAIDs), strong opioid analgesics, bisphosphonates and monoclonal antibodies targeted to the inhibition of osteoclastic activity are the main drug treatments for breast cancer-induced bone pain (BCIBP) (Kane et al., 2015). However, these treatments may be inadequate and/or may evoke dose-limiting side effects (Cleeland et al., 2003; Sang and Bennett, 2009; Dib-Hajj and Waxman, 2014; Shenoy et al., 2016). Hence, it is important to identify new targets for development of novel analgesic agents with improved efficacy and tolerability for alleviation of BCIBP. One such target is the somatostatin receptor type 4 (SST4 receptor) (Crider and Witt, 2007; Somvanshi and Kumar, 2014; Abdel-Magid, 2015). J-2156 [(1′S,2S)-4amino-N-(1′-carbamoyl-2′-phenylethyl)-2-(4″-methyl-1″-naphthalenesulfonylamino)butanamide] is an agonist that binds with nanomolar affinity to the human SST4 receptor and that has more than 300-fold selectivity compared with other somatostatin receptors (Engström et al., 2005). Although there are several known ligands/agonists of the SST4 receptor (Erchegyi et al., 2003a,b; Grace et al., 2003; Crider and Witt, 2007), J-2156 has high potency and a low propensity to cause receptor desensitization (Engström et al., 2005, 2006). Additionally, in work by others, J-2156 has been shown to induce pain relief in animal models of both inflammatory and neuropathic pain (Sándor et al., 2006; Schuelert et al., 2015).

However to date, the efficacy of J-2156 in BCIBP, has not been assessed preclinically. Hence this study was primarily designed to assess the efficacy of J-2156 to alleviate mechanical allodynia and mechanical hyperalgesia in a rat model of BCIBP previously validated in our laboratory (Shenoy et al., 2017). To complete the preclinical profile of J-2156, we also tested its *in vitro* potency and selectivity toward human and rat SSTR4 receptor as well as a panel of 67 known pharmacological targets. Due to limited permeability of the blood-brain barrier at the doses tested, J-2156 is considered likely to act on peripheral SST4 receptors, although it is also capable of inhibiting spinal neurons (Schuelert et al., 2015). Peripheral small diameter peptidergic and non-peptidergic C-fibers as well as medium-large diameter fibers including A-δ and A-β fibers have key roles in the neural signaling of cancer pain (Urch et al., 2003; Donovan-Rodriguez et al., 2005; Mantyh, 2006; Mao-Ying et al., 2006; Colvin and Fallon, 2008; Ye et al., 2014). Our work herein is the first to assess the distribution of the SST4 receptor in primary somatosensory neurons of the ipsilateral lumbar dorsal root ganglia (DRGs) of rats in the BCIBP model. In addition, in the same animal model, we have assessed the effect of J-2156 on lumbar spinal dorsal horn expression levels of phosphorylated extracellular signal-regulated kinase (pERK), a protein implicated in the pathobiology of central sensitization and persistent pain (Gao and Ji, 2009).

## Materials and methods

### Drugs, chemicals, and reagents

Triton^TM^ X-100, Tween 20 and paraformaldehyde (PFA) were purchased from Sigma-Aldrich® (NSW, Australia). Isoflurane (IsoFlo^TM^) was purchased from Abbott Australasia Pty Ltd., (NSW, Australia). Medical oxygen was purchased from Coregas Pty Ltd., (NSW, Australia). Triple antibiotic powder (Tricin®) was purchased from Jurox Pty Ltd., (NSW, Australia). Benzylpenicillin (BenPen^TM^, benzylpenicillin sodium for injection) was purchased from CSL Ltd., (VIC, Australia). Pentobarbitone (Lethabarb®, pentobarbitone sodium) was purchased from Virbac (Australia) Pty Ltd., (NSW, Australia). Eye ointment (Refresh Night Time®) was purchased from Allergan Australia Pty Ltd., (NSW, Australia). 4′,6-diamidino-2-phenylindole, dihydrochloride (DAPI), Prolong® Gold antifade reagent, phosphate-buffered saline (PBS), medium 199 (1X), horse serum, Dulbecco's phosphate-buffered saline (DPBS, 1X) and 0.25% trypsin-EDTA (1X) were purchased from Thermo Fisher Scientific Australia Pty Ltd., (VIC, Australia). Normal goat serum (NGS) was purchased from Cell Signaling Technology® (MA, USA). Tissue-Tek® O.C.T. Compound was purchased from ProSciTech Pty Ltd., (QLD, Australia). Sodium Chloride injection BP (British Pharmacopeia) (0.9%) was purchased from Pfizer Australia Pty Ltd., (NSW, Australia). J-2156 was obtained from Boehringer Ingelheim Pharma GmbH & Co. KG, (BW, Germany).

### Assessment of molecular selectivity, affinity, and potency of J-2156

#### Assessment of reactivity of J-2156 to receptors of the somatostatin family

Binding studies were conducted to determine the selectivity and affinity of J-2156 to human somatostatin receptor types 1–5 and to the rat SST4 subtype. Radioligand binding assays were performed in 96-well ELISA plates (NUNC, Denmark) using binding buffer (10 mM/L HEPES; 1 mM/L EDTA; 5 mM/L MgCl_2_x6H_2_0) containing 30 μg/mL bacitracin (Sigma, Germany), and 5 mg/ml protease-free BSA fraction V (Sigma, Germany, A-3059). The pH was adjusted to 7.6 using 4 M NaOH. Selectivity of J-2156 was determined using membrane preparations from CHO-K1 cells stably-expressing human somatostatin receptor types 1–5 and rat somatostatin receptor type 4. CHO-K1 cell membranes expressing human somatostatin receptor type 1 (ES-520-M400UA, with 40 ug/well concentration), type 2 (ES-521-M400UA, with 25 ug/well concentration), type 3 (ES-522-M400UA, with 1.5 ug/well concentration), and type 5 (ES-522-M400UA, with 25 ug/well concentration) were procured from Perkin Elmer, Waltham, MA. CHO-K1 cell membranes expressing human somatostatin receptor type 4 were procured from BioTrend, Germany (with 0.5 ug/well concentration). CHO-K1 cell membranes expressing rat somatostatin receptor type 4 were procured from Perkin Elmer, Waltham, MA (with 200 ug/well concentration). J-2156 was tested in duplicate, over a range of concentrations from 10^−12^ to 10^−5^ M and the endogenous ligand somatostatin 14 (BioTrend, Germany) was run in parallel as a positive control. Octreotide was used as a negative control for the SST4 receptor, as it is known to exhibit affinity for subtypes 2, 3, and 5, with low affinity for subtypes 1 and 4 (Patel, 1999). Octreotide stock solution was 10 mM concentration, and was procured from Aneo systems, Germany, SP010100B. Binding curves were derived from competition experiments against 0.05 nM [125I]-Tyr3-somatostatin-(1–14) (ANAWA Trading SA, Switzerland). The final activity of the label was 80.5 TBq/mM. The end volume was 250 μl/well, where initially 25 μl of compound was added to each well, followed by 25 μl of radioligand and then finally 200 μl of cell suspension. Total-binding was defined using only the assay buffer and nonspecific binding was defined with 1 μM somatostatin 14. The initial incubation was carried out at room temperature (23–4°C) for 3 h with constant shaking. The reaction was then terminated by rapid filtration through a Packard harvester (Perkin Elmer, Waltham, MA) onto unifilter-96 GF/B filter plates (Perkin Elmer, Waltham, MA) which had been pre-soaked in 0.3% polyethyleneimine (Sigma, Germany). The plates were washed 3 times using ice-cold (4°C) physiological (9 g/L) sodium chloride (NaCl) solution (Merck, USA) at an approximate volume of 300 μL/well. Following addition of 50 μL/well of scintillant (Microscint 20, Packard, USA), the plates were further incubated at room temperature (23–24°C) for 1 h in the dark. Analysis for radioactivity was conducted using the Top Count NXT^TM^ microplate scintillation counter (Packard, USA).

#### Assessment of binding of J-2156 to other pharmacological targets

J-2156 was assessed in a lead profiling binding assay screen (Ricerca Biosciences LLC) consisting of 67 pharmacological targets, to identify the binding of J-2156 for a range of receptors, ion channels and transporters, by following standard radioligand binding assay protocols (Guerrero et al., 2010a,b; Strøbæk et al., 2013). These included important targets like sodium ion channels because they are known to be involved in pain pathophysiology (Amir et al., 2006; Dib-Hajj et al., 2009, 2010). The significance level of inhibition or stimulation of binding at 10 μM was predefined at ≥50% for all the assays.

#### Potency of J-2156: cAMP inhibition

Total cAMP accumulation was measured using the LANCE cAMP detection kit (Perkin Elmer, Waltham, MA), in 384 optical assay plates. J-2156 was tested at concentrations ranging from 10^−12^ to 10^−5^ M at a volume of 2 μL/well. All dilutions were prepared in stimulation buffer prepared from HBSS 1x solution (Gibco, UK), with an addition of 5 mM HEPES buffer (Gibco, UK), 0.1% BSA (Serva, Germany) and 500 mM isobutylmethylxanthine (IBMX, Sigma, Germany). Each concentration of standard or J-2156 was tested in duplicate or triplicate, respectively. The cAMP standard was prepared using the standard solution (Perkin Elmer, Waltham), to make an end concentration of 1 μM. Serial dilutions were then prepared, using the stimulation buffer, from 1,000 to 0.01 nM, of cAMP standard. Initially the compounds or standards were pipetted to each well. Intact H4 cells expressing either human or rat SST4 receptors (Perkin Elmer, Waltham) were used at 1250 cells/well or 1000 cells/well respectively with a volume of 5 μl/well. The cells were stored in frozen aliquots (1 ml) at −80°C and defrosted on the day of use. The vial was suspended in 9 ml DPBS solution and the number of cells were counted using 0.4% trypan blue stain (Invitrogen, Life Technologies, Germany) and the Countess automated cell counter (Invitrogen, Life Technologies, Germany). A pellet was formed by centrifuging the cell suspension at 1,200 rpm for 5 min, the DPBS was aspirated off and the pellet was re-suspended in the calculated volume of assay stimulation buffer. To this, the supplied Alexa Fluor® antibody was added at a dilution of 1:100. Next, the cells were added to the plate to allow pre-treatment with the compounds for 10 min at room temperature (23–24°C). For the standard, 5 μL of the supplied Alexa Fluor® antibody was mixed with 495 μL of stimulation buffer and rather than adding cells, 5 μL of the antibody solution was added to the standard wells. After 10 min, stimulation was achieved by adding either 10 or 30 μM forskolin (Sigma, Germany) for human or rat receptors, respectively. The final volume was 10 μL/well. Plates were incubated for 1 h at room temperature (23–24°C) with constant shaking. Finally, the detection buffer (Perkin Elmer, Waltham) was added (10 μL/well) and the plates were centrifuged at 1,000 rpm. This was followed by a further incubation period of 1 h at room temperature (23–24°C) in the dark. Plates were read using an EnVision Xcite 2104 multilabel reader (Perkin Elmer, Waltham, MA) at 665 nm.

### Cell culture

Walker 256 cells were used to induce BCIBP in rats as reported in the literature (Shenoy et al., 2016). The Walker 256 breast cancer cell line [LLC-WRC 256 (ATCC® CCL-38^TM^)] at passage number 290 was purchased from the American Type Culture Collection (ATCC; VA, USA). The cells were cultured and passaged following the ATCC guidelines. The cells were thawed from frozen stocks and cultured in 75 cm^2^ Cellstar® flasks (Greiner bio-one) at 37°C (5% CO2: 95% atmospheric air) in 20 mL of Medium 199 (1X) with an additional supplementation of 5% horse serum. In order to detach the cells, they were gently rinsed with 3 mL of DPBS (1X), followed by treatment with 2 mL of 0.25% trypsin-EDTA (1X). The cells thus detached were harvested by centrifuging with 8 mL of medium for 4 min at 200 × g. The supernatant thus obtained was discarded, the pellet re-suspended in 3 mL of DPBS and cell counting was performed with a hemocytometer. After again centrifuging the pellet for 4 min at 200 × g, the cells were suspended in DPBS to obtain a final dilution of 4 × 10^5^ cells/10 μL. Heat-killed Walker 256 cells were prepared similarly to the live cells, however, the cells were heated to 90°C for 15 min.

### Animals

Animals were purchased from the Herston Medical Research Centre (Brisbane, Australia) of The University of Queensland. A total of 45 female Wistar Han (HsdBrlHan) rats were used in this study. At the time of arrival in our research facility, the rats were ~3–4 weeks old and their body weights were in the range ~50–70 g. Rats were housed in small groups of two to three in individually ventilated cages in a room having a controlled temperature (23 ± 2°C) and a 12 h/12 h light–dark cycle. Standard rodent chow (Specialty Feeds, WA, Australia) and tap water were available to all rats *ad libitum*. Kimwipes (Kimberly-Clark Professional, NSW, Australia) and Rat Chewsticks (Able Scientific, WA, Australia) were provided in the individual cages as environmental enrichment. Rats were subject to acclimatization in the animal housing facility for at least 3 days prior to initiating experiments. The experiments on these rats were performed in the light phase. The procedures involving animal experimentation were approved by the Animal Ethics Committee of The University of Queensland (QLD, Australia). The experiments described herein were performed as per the requirements of the Australia Code of Practice for the Care and Use of Animals for Scientific Purposes (8th edition, 2013).

### Surgical procedure

Unilateral intra-tibial injections (ITI) were performed in accordance with the procedures reported previously (Mao-Ying et al., 2006; Muralidharan et al., 2013), with some modifications. In brief, rats in the weight range ~80–120 g were anesthetized deeply using 3% isoflurane delivered in oxygen. To avoid drying of the eyes during the surgical procedure, eye ointment was applied. Benzylpenicillin (60 mg per rat) injection was subcutaneously administered. A rostro-caudal incision of ~1 cm was made on the upper medial half of the lower left hindlimb. Following exposure of the tibia, using a 23-gauge needle, a hole was drilled into the bone below the knee joint, medial to the tibial tuberosity. Either live (BCIBP group) or heat-killed (sham-injected group) Walker 256 cells (4 × 10^5^ cells in 10 μL DPBS) were injected into the bone cavity with a Hamilton® syringe (80508:705SN 50 μL SYR SPECIAL (22/2″/4), NV, USA). Subsequently, the drilled hole in the bone was sealed immediately with Ethicon^TM^ W810 bone wax (Johnson-Johnson International, Diegem, Belgium). The muscles and the skin were subsequently sutured using non-absorbable USP 5/0 Dysilk® suture (Dynek Pty Ltd., SA, Australia). Finally, topical antibiotic powder was applied to the stitched wound. The surgical procedure was minimally invasive such that chipping or cracking of bone was avoided. The hindlimb injected with cells is termed the “ipsilateral” hindlimb and the non-injected hindlimb is termed the “contralateral” hindlimb. Following completion of the surgical procedure, rats were closely monitored for recovery and their general health was routinely assessed at least once per week until the end of the study. Each rat was carefully assessed for their appearance, tonic movements, clonic movements, gait, stereotypy and any bizarre behavior. The method of observation of clinical parameters was modified from previously established standard methods (Moser and MacPhail, 1990; Haggerty, 1991) and Guidance Document on the Recognition, Assessment, and Use of Clinical Signs as Humane Endpoints for Experimental Animals Used in Safety Evaluation (OECD Environmental Health and Safety Publications Series on Testing and Assessment No. 19, 2000). The details of the clinical parameters assessed and a template of the symptoms monitoring sheet used have been provided in our previous report (Shenoy et al., 2017).

### Behavioral studies

#### Assessment of mechanical allodynia in the hindpaws

Development of mechanical allodynia in the bilateral hindpaws was assessed using calibrated von Frey filaments (Stoelting Co., Wood Dale, IL, USA). The lowest mechanical threshold which elicited a paw withdrawal response was measured (Ren, 1999). Mechanical allodynia was measured in terms of changes in paw withdrawal thresholds (PWTs) in agreement with other studies (Bennett, 2010; Almarestani et al., 2011; Dong et al., 2011; Ke et al., 2013; Bao et al., 2014; Bu et al., 2014; Xia et al., 2014). Rats were individually placed in wire mesh cages and acclimatized for ~15–30 min before applying the von Frey filaments. The filaments were individually applied to the surface of the plantar region of each hindpaw until they were slightly buckled. If there was no response after 3 s of applying the filament, the next higher filament was used in the ascending order (2, 4, 6, 8, 10, 12, 14, 16, 18, and 20 g) until the response was observed. If the hindpaw withdrawal response was observed within 3 s the next filament evoking a lower force was used. Initiation of the testing was done using a 6 g filament, and the subsequent force was modified depending upon the previous response. The baseline PWTs of the ipsilateral as well as the contralateral hindpaws were recorded thrice at an interval of 5 min each and the mean of these readings was calculated. For behavioral testing after administration of J-2156 at pre-defined intervals over a 3 h period, the starting filament depended on the previous response. Rats having ipsilateral PWTs ≤ 6 g were defined as having fully developed mechanical allodynia. All the assessments using von Frey filaments were performed by an investigator blinded to the treatment.

#### Assessment of mechanical hyperalgesia in the hindpaws

Development of mechanical hyperalgesia in the hindpaws was tested using an Analgesy-meter (Ugo Basile, Italy). The mechanical force required to produce a withdrawal response in each of the hindpaws (paw pressure thresholds, PPTs) of each rat was assessed (Randall et al., 1957). Mechanical hyperalgesia was measured in terms of changes in PPTs as per the common practice (Whiteside et al., 2004; Al-Rejaie et al., 2015; Griggs et al., 2016; Ferrari et al., 2017; Zambelli et al., 2017). Briefly, each hindpaw was individually placed on a small plinth beneath a cone-shaped pusher with a rounded tip so as to avoid damage to the hindpaw tissue. The mechanism of exerting the force was started by depressing the pedal-switch. The pedal was released immediately when the rat began to struggle and the applied force was subsequently noted. A maximum cut-off force of 200 g was used to prevent injury to the hindpaws. Three baseline readings for each of the hindpaws were recorded with an interval of 5 min between successive readings. The mean baseline PPTs were calculated for each hindpaw. For pharmacological assessment of the anti-hyperalgesic effect of J-2156, rats with an ipsilateral PPT ≤ 80 g were considered to have fully developed mechanical hyperalgesia. All the PPT assessments were performed by an investigator blinded to the treatment group.

### Administration of J-2156 and behavioral testing

Dosing of animals was performed by one investigator while the behavioral assessments were subsequently performed by a second investigator blinded to the treatment groups to ensure that investigator bias was kept to a minimum throughout the procedure. Based on the animal ethics principle of “Reduction,” a “washout protocol” was used such that each rat received up to a maximum of four individual intraperitoneal (i.p.) doses of J-2156 or vehicle with at least 2–3 days of “washout” between successive doses as per the common practice in pain research (Zeppetella et al., 2003; Kim et al., 2010; Otto et al., 2011; Varamini et al., 2012; Muralidharan et al., 2013, 2017; Han et al., 2014; Khan et al., 2014; Otis et al., 2016; Friton et al., 2017a,b; Shenoy et al., 2017; Suzuki et al., 2017). The animals that received a new treatment on a different occasion after the washout period, were cumulatively added to build the “n” numbers. Sodium Chloride injection BP (0.9%) was used as vehicle for preparing J-2156 dosing solutions. Rats with fully developed hindpaw hypersensitivity were administered a single bolus dose of J-2156 (1, 3, and 10 mg/kg, i.p.) or vehicle between day 7 and 14 post-ITI of Walker 256 cancer cells. PWTs or PPTs were assessed in both the hindpaws immediately before J-2156 or vehicle i.p. administration and subsequently at 0.25, 0.5, 0.75, 1.0, 1.25, 1.5, 2, and 3 h post-dosing.

### Immunohistochemistry

Rats were euthanized on day 7 post-ITI with an injection of pentobarbitone and immediately perfusion-fixed with 4% PFA. The volume of 4% PFA used to fix one animal was around ~150 mL with an intra-cardiac flow rate of ~15 mL/min for ~10 min. The tissues (tibiae/brain/liver/spinal cord/DRGs) were harvested and further post-fixed with 4% PFA for ~3 h. In the liver, somatostatin receptors type 1, 2, 3, and 5 are expressed, but expression levels of the SST4 receptor are negligible (Murray et al., 2004; Reynaert et al., 2004; Song et al., 2004; Jung et al., 2006). Conversely, the SST4 receptor is abundantly expressed in brain tissue (Selmer et al., 2000a,b). Hence, coronal sections of brain and sections of liver were used as positive and negative controls in the validation process, respectively. The spinal cord tissues used to assess the effect of J-2156 on pERK levels, were collected from the rats at the time of peak effect of J-2156. The tissues were cryoprotected successively in 15% sucrose/PBS and 30% sucrose/PBS at 4–8°C and then immersed in a 1:1 mixture of OCT:30% sucrose/PBS at 4–8°C, followed by freeze-mounting in Tissue-Tek® O.C.T. Compound. Frozen coronal sections of brain and transverse sections of liver, lumbar L4-6 spinal cord and lumbar L4-6 DRGs (7 μm thick) were obtained using a Cryostar NX70, (Thermo Fisher Scientific, Waltham, USA) and mounted on Uber Plus charged slides (InstrumeC Pty Ltd., VIC, Australia). Lumbar L4-6 segment was chosen, because the tibial nerve is innervated by the lumbar L4-6 spinal level in rats (Romano et al., 1991). The sections were washed with PBS (pH 7.4) solution thrice for 5 min each, and blocked with 10% NGS in PBS containing 0.3% Triton^TM^ X-100 for 1–2 h at 23 ± 2°C. Further, these sections were incubated with the respective primary antibody (or a combination of primary antibodies for co-localization experiments), diluted in 2% NGS in PBS containing 0.1% Tween 20, overnight at 4–8°C. The primary antibodies used in the present study were anti-SST4 receptor antibody PA3208 (1:250 dilution, Life Technologies Australia Pty Ltd., VIC, Australia), anti-somatostatin antibody ab183855 (1:500 dilution, Abcam, VIC, Australia), anti-substance P (SP) antibody ab14184 (1:25 dilution, Abcam, VIC, Australia), anti-neurofilament 200 kDa (NF200) antibody ab82259 (1:50 dilution, Abcam, VIC, Australia) and anti-pERK antibody 4370S (1:50 dilution, Cell Signaling Technology®, MA, USA). Isolectin B4 (IB4) L 2895 (1:100 dilution, Sigma-Aldrich®, NSW, Australia) was also used. We used SP, IB4 and NF200 as specific somatosensory cell markers for peptidergic C-fibers, non-peptidergic C-fibers and medium-large diameter fibers including A-δ and A-β fibers, respectively (Le Pichon and Chesler, 2014). The sections were then washed twice for 5 min each with PBS containing 0.1% Tween 20 and once for 5 min with PBS. These sections were further incubated with the corresponding secondary antibody (or a combination of secondary antibodies for co-localization experiments), diluted in PBS containing 0.1% Tween 20, for 2 h at 23 ± 2°C in the dark (~0.002 lux). The secondary antibodies used in the present study were goat anti-mouse IgG (H+L), Alexa Fluor 546 A-11030 (1:600 dilution, invitrogen^TM^, OR, USA), goat anti-mouse IgG (H+L), Alexa Fluor 488 A-11029 (1:500 dilution, invitrogen^TM^, OR, USA), goat anti-rabbit IgG (H+L), Alexa Fluor 488 A-11034 (1:600 dilution, invitrogen^TM^, OR, USA), goat anti-rabbit IgG (H+L), Alexa Fluor 546 A-11035 (1:1,000 dilution, invitrogen^TM^, OR, USA) and Cy™ 3 AffiniPure goat anti-rabbit IgG (H+L) 111-165-003 (1:600 dilution, Jackson ImmunoResearch Inc., PA, USA). The sections were then washed twice with PBS containing 0.1% Tween 20 and once with PBS for 5 min each. The sections were subsequently incubated with DAPI (0.5 μg/mL solution) for around 5–10 min and finally washed with PBS twice for 5 min each. The cover-slips were mounted on the sections along with Prolong® Gold antifade reagent. The mounted slides thus obtained were set aside to dry and stabilize in the dark at 4–8°C overnight. Finally, the images were captured with a fluorescence microscope and analyzed. Assessment of the presence of Walker 256 cancer cells in the tibiae of rats was performed using anti-Cytokeratin 18 antibody [C-04] ab668 (1:100 dilution, Abcam, VIC, Australia) by following the protocol described previously (Shenoy et al., 2017).

### Acquisition of images and analysis

Images of experiments from immunohistochemistry were captured with an Axioskop 40 microscope that was attached to an Axiocam MRm camera. The images were processed using AxioVision Rel. v4.8 software (imaging equipment and software were from Carl Zeiss, Göttingen, Germany). For each of the experiments, images were captured at a fixed exposure time, which was optimized using auto-exposure settings of AxioVision Rel. v4.8 software. The filters used in the microscope were chosen to suit the respective fluorophore of the secondary antibody used in each of the experiments. For quantitative analysis, at least 3–4 non-adjacent sections per animal from each of the groups (*n* = 3–4/group) were randomly selected. Images were assigned codes by the first investigator and quantitative analysis was performed by the second investigator in a blinded manner. Densitometric counts were quantified using the built-in tool “AutoMeasure” of the AxioVision Rel. v4.8 software. During this process, a uniform threshold setting was used to create binary images and precisely analyse the signals in the identical regions of interest. We used the inbuilt template facility in the software to obtain the bright field view in order to locate the laminae I-IV of spinal dorsal horns for the purposes of immunohistochemical assessments. Laminae I-IV of spinal dorsal horn were chosen as these are the layers that correspond with the bone originating pain inputs (Sah et al., 2003; Le Pichon and Chesler, 2014; Nencini and Ivanusic, 2016). The densitometric counts obtained for each image were grouped according to the treatment groups and the data were expressed as fold-changes in fluorescence intensity, relative to the respective control group (Han et al., 2014; Muralidharan et al., 2014; Khan et al., 2015).

### Data analysis and statistical analysis

All values have been expressed as mean ± standard error of the mean (SEM). In the radioligand binding assays of J-2156 with somatostatin receptors, Ki denotes a dissociation/inhibition constant, and can be considered to be the reciprocal of the binding affinity (Cheng and Prusoff, 1973). The Cheng-Prusoff equation was used to derive the Ki values (Cheng and Prusoff, 1973). In the cAMP inhibition assays, the term IC_50_ denotes the concentration producing 50% inhibition (Cheng and Prusoff, 1973). The IC_50_ values were reported as the concentration of the compounds producing 50% inhibition of the forskolin-stimulated cAMP production. The affinity and potency data were calculated using GraphPad Prism (v6.00) software.

All other graphs were stored, represented and analyzed using the GraphPad Prism^TM^ (v7.00) software. The ED50 values were calculated as per the method described previously (Rowlett et al., 1999; Muralidharan et al., 2013, 2017; Han et al., 2014; Khan et al., 2014; Shenoy et al., 2017). The change in the PWT values (ΔPWT) in the hindpaws of rats following administration of single bolus doses of J-2156 or vehicle was determined by subtracting the predosing PWT values from the respective post-dosing PWT values for individual rats at each time-point i.e.,

$$\begin{array}{l} \Delta \text{PWT value}=\left(\text{postdosing PWT}\right)-\left(\text{predosing baseline PWT}\right) \end{array}$$

Area under the curve (AUC) of ΔPWT vs. time graphs (ΔPWT AUC values) for individual rats was calculated using trapezoidal integration to find the extent and duration of the anti-allodynic effect of each dose of J-2156. Next, the ΔPWT AUC values were further converted to a percentage of the maximum possible ΔPWT AUC (% MAX ΔPWT AUC) by using the following formula:

$$\begin{array}{l} \%\text{MAX }\Delta \text{PWT AUC}=\frac{\Delta \text{PWT AUC }\times \;100}{\text{Maximum }\Delta \text{PWT AUC}} \end{array}$$

Dose–response curves were produced by plotting mean (± SEM) % MAX ΔPWT AUC values vs. log dose of J-2156. The ED_50_ value (dose at which the test-item produced 50% of its anti-nociceptive effect) for J-2156 was calculated by using non-linear regression (an iterative curve-fitting technique for sigmoidal dose-response functions) in GraphPad Prism™ v7.00. The PPT data were processed using a similar procedure as described above for the PWT data.

GraphPad Prism^TM^ v7.00 software was used to perform statistical analyses. The criterion for statistical significance was *p* ≤ 0.05. To assess the between-group differences of baseline PWT/PPT values and PWT/PPT values following J-2156 administration, two-way analysis of variance (ANOVA) followed by the Bonferroni test was used. To assess differences between protein (SST4 receptor, somatostatin and pERK) expression in neural tissues as well as the data from co-localization experiments, the Mann-Whitney test was used. For statistical comparisons using ANOVA, the F values are reported along with their associated degrees of freedom (treatment, time, interaction, and residual). For two-way ANOVA, F values are reported as F(df of treatment, time, interaction/residual).

## Results

### Assessment of molecular selectivity, affinity, and potency of J-2156

**Figure 1:**
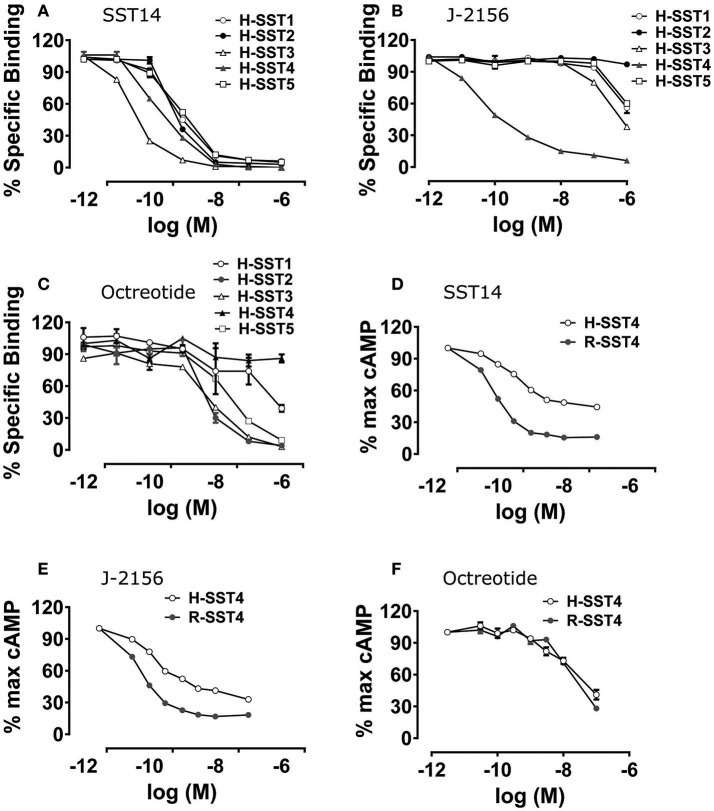
Binding affinity and potency of J-2156 at the five somatostatin receptor types. Panels in the figure show **(A)** binding curves of the endogenous somatostatin receptor ligand- somatostatin 14; no selectivity was observed, **(B)** binding curves of J-2156; more than ~300-fold selectivity for the SST4 receptor was observed relative to that for the other somatostatin receptor types, **(C)** binding curves of octreotide; low affinity was seen at SST4 receptor. Competition binding assays were run using ^125^I radiolabelled somatostatin 14 (0.05 nM) in the presence of 0.5 μg protein. **(D,E)** present the concentration response curves showing that SST14 and J-2156 inhibited forskolin (30 μM and 10 μM for rat and human SST4 receptor, respectively)-stimulated cAMP production in H4 cells expressing the rat and the human SST4 receptor types. Similarly, **(F)** shows that octreotide, the negative control, was unable to inhibit cAMP production in both human and rat SST4 receptor. H-SST1-5, human somatostatin receptor types 1-5; R-SST4, rat somatostatin receptor type 4.

The selectivity and binding affinity of various somatostatin agonists for the five human somatostatin receptor types (SST1-5) and the rat SST4 type were established using radioligand binding assays. Radioligand binding assays assessed the binding of J-2156 and other ligands in the cell membrane preparations, while cAMP assays were performed using intact functioning cells. The somatostatin receptor selective endogenous ligands, somatostatin 14 and somatostatin 28, as well as the non-selective endogenous ligand, corticostatin 17, were used as positive controls. The affinities of J-2156 and these endogenous ligands for all somatostatin receptor types are summarized in Table 1. While the profile of the endogenous ligand somatostatin-14 was non-selective between somatostatin receptor types (Figure 1A), J-2156 displayed a strong affinity in the nanomolar range for the SST4 receptor only, with low affinity for the other somatostatin receptor types (Figure 1B). Octreotide, used as negative control for SST4 receptor, had nanomolar affinity for human somatostatin receptor types 2, 3, and 5, suggesting selectivity to these receptor subtypes; whereas low affinity was seen at the human SST4 receptor (Figure 1C). Similarly, octreotide had low affinity for the rat SST4 receptor. Thus, we confirmed that J-2156 is a SST4 receptor-selective agonist with over ~300-fold selectivity for this receptor compared to other receptor types of the somatostatin family, consistent with a previous report by others (Engström et al., 2005).

**Table 1:** Selectivity of somatostatin agonists at human somatostatin receptor types 1–5 and the rat somatostatin receptor type 4.

**Compound**	**Mean (±SEM) Ki values (nM)**					
	**H-SST1 receptor**	**H-SST2 receptor**	**H-SST3 receptor**	**H-SST4 receptor**	**H-SST5 receptor**	**R-SST4 receptor**
Somatostatin 14	0.54 ± 0.02	2.14 ± 0.32	0.07 ± 0.01	0.28 ± 0.04	0.66 ± 0.04	0.38 ± 0.05
Somatostatin 28	0.14 ± 0.10	2.81 ± 0.04	0.06 ± 0.01	0.36 ± 0.09	0.52 ± 0.03	1.48 ± 1.39
Corticostatin 17	4.79 ± 1.12	33.88 ± 0.33	2.45 ± 0.10	2.51 ± 0.35	5.49 ± 0.64	0.91 ± 0.55
J-2156	588.8 ± 266	*25, 118.9*±*4, 781.2*	234.4 ± 112.5	0.56 ± 0.10	467.7 ± 155.9	0.89 ± 0.64
Octreotide	211.75 ± 7.55	4.90 ± 0.73	6.63 ± 0.32	*84, 969.9*±*8, 098.88*	20.86 ± 2.58	179.4 ± 44.5

*H-SST1-5, human somatostatin receptor types 1-5; R-SST4, rat somatostatin receptor type 4. n = 3–6*.

To further characterize the selectivity profile, J-2156 was tested against a standard panel of 67 targets (including known pharmacologically relevant G-protein coupled receptors, ion channels and transporters; Ricerca Biosciences LLC) at a single concentration of 10 μM. J-2156 did not significantly stimulate or inhibit any of the pharmacological targets at/above the predefined significance level of ≥50%. Minor partial modulation was observed for a few targets ranging from 20 to 35%. However, at the doses found to be efficacious in the present study (3–10 mg/kg; i.p.), the peak plasma concentration is expected to be between 300 and 1,000 nM (data extrapolated from in house pharmacokinetic studies done by Boehringer Ingelheim personnel in satellite Wistar Han naive rats) (Schuelert et al., 2015). Hence, the binding of J-2156 on these non-cognate pharmacological targets can be considered to be very low and pharmacologically negligible. The data showing J-2156 mediated non-cognate stimulation or inhibition of all these targets have been summarized in Supplementary Table 1. These results show that J-2156 has no significant cross-reactivity with other pharmacological targets and is highly selective for the SST4 receptor only.

To confirm that J-2156 functionally activates the SST4 receptor, forskolin-stimulated cAMP assays were conducted. We found that J-2156 is a potent full agonist at both human and rat SST4 receptors (Table 2). We observed that J-2156 was even more potent than that of the endogenous ligands of the SST4 receptor, in support of the notion that J-2156 is a “superagonist,” as originally claimed by its discoverers (Engström et al., 2005). The potencies of SST14 toward human and rat SST4 receptor were quite similar (Figure 1D), and so were the potencies of J-2156 toward human and rat SST4 receptors (Figure 1E). The observed difference of <2-fold was observed in the potencies of J-2156 between the two species. Octreotide was unable to inhibit cAMP production, indicating low potency for both the human and rat SST4 receptor (Figure 1F). The results also confirmed that the SST4 receptor can functionally couple to the adenylyl cyclase pathway potently.

**Table 2:** Potency of somatostatin agonists for human and rat SST4 receptors determined by forskolin-stimulated cAMP functional assays.

**Compound**	**Mean (±SEM) IC_50_ values (nM)**	
	**H-SST4**	**R-SST4**
Somatostatin 14	0.19 ± 0.08	0.16 ± 0.005
Somatostatin 28	0.79 ± 0.10	0.19 ± 0.04
Corticostatin 17	3.16 ± 0.44	1.00 ± 0.06
J-2156	0.05 ± 0.01	0.07 ± 0.005
Octreotide	2, 098.74 ± 420.37	1, 798.04 ± 307.56

*H-SST4, human somatostatin receptor type 4; R-SST4, rat somatostatin receptor type 4. n = 3*.

The data from radioligand binding assays and cAMP inhibition assays overall suggest that J-2156 not only has high potency, but it also displays high selectivity toward the SST4 receptor. Hence, J-2156 was used as a valid preclinical tool compound to investigate the *in vivo* role of the SST4 receptor in this rat model of BCIBP.

### Assessment of cancer cells in tibiae

**Figure 2:**
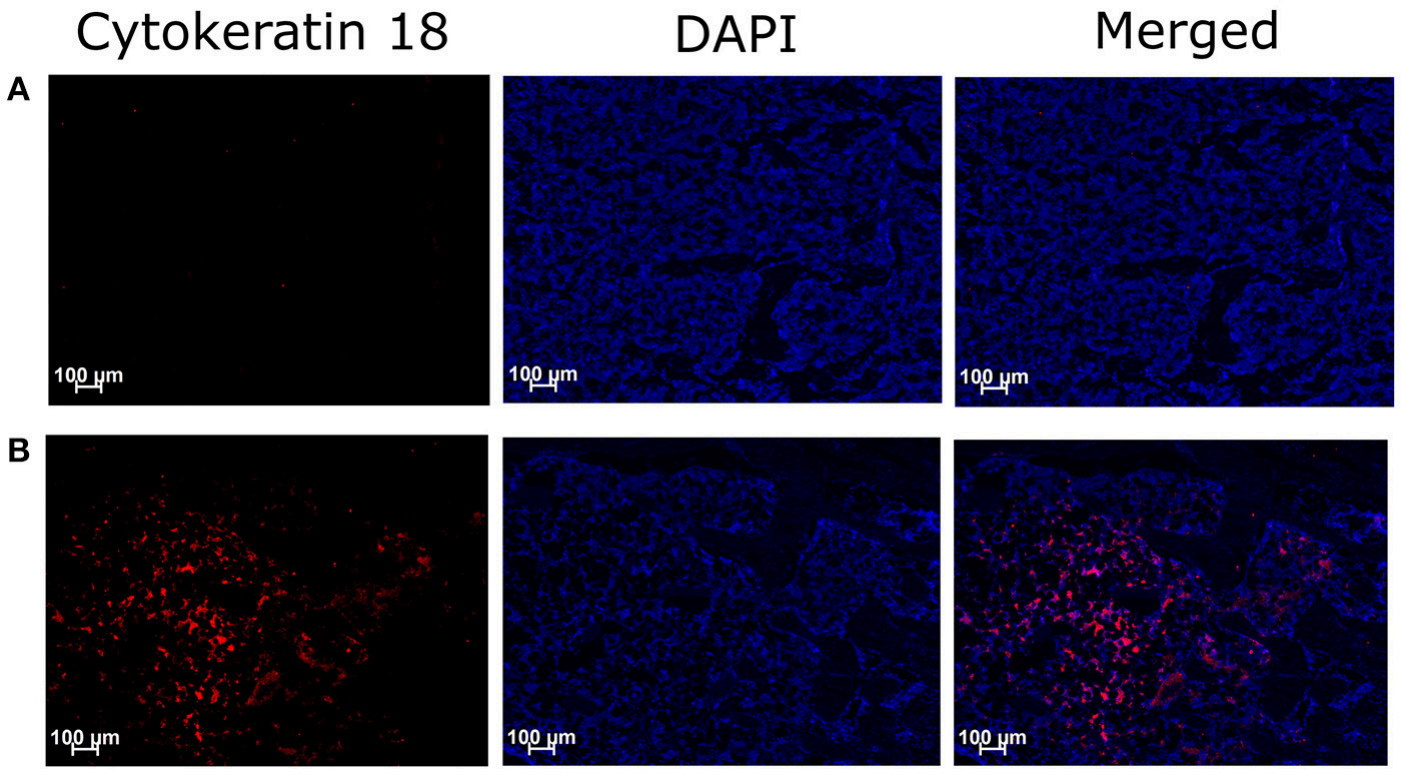
Immunohistochemical staining of Cytokeratin 18 in tibial sections of BCIBP rats. Panels in the Figure show immunofluorescence images of a representative tibial section of **(A)** a sham rat and **(B)** a BCIBP rat.

Immunohistochemical assessment of longitudinal frozen sections of tibiae from BCIBP rats against Cyokeratin 18 confirmed the presence of Walker 256 cancer cells in the BCIBP group (Figure 2). However, this immunofluorescence was absent in the corresponding sections of tibiae from sham rats, as expected.

### Development of mechanical allodynia and mechanical hyperalgesia in the bilateral hindpaws

All animals that received a unilateral ITI of live Walker 256 cells developed pain behaviors, with no significant health issues being observed. None of the rats were excluded from the present study. Unilateral ITI of Walker 256 cells induced temporal development of mechanical hypersensitivity in the bilateral hindpaws in rats, in agreement with our previous study (Shenoy et al., 2017) and findings by others (Mao-Ying et al., 2006, 2012).

### Anti-allodynic effect of J-2156 in BCIBP

**Figure 3:**
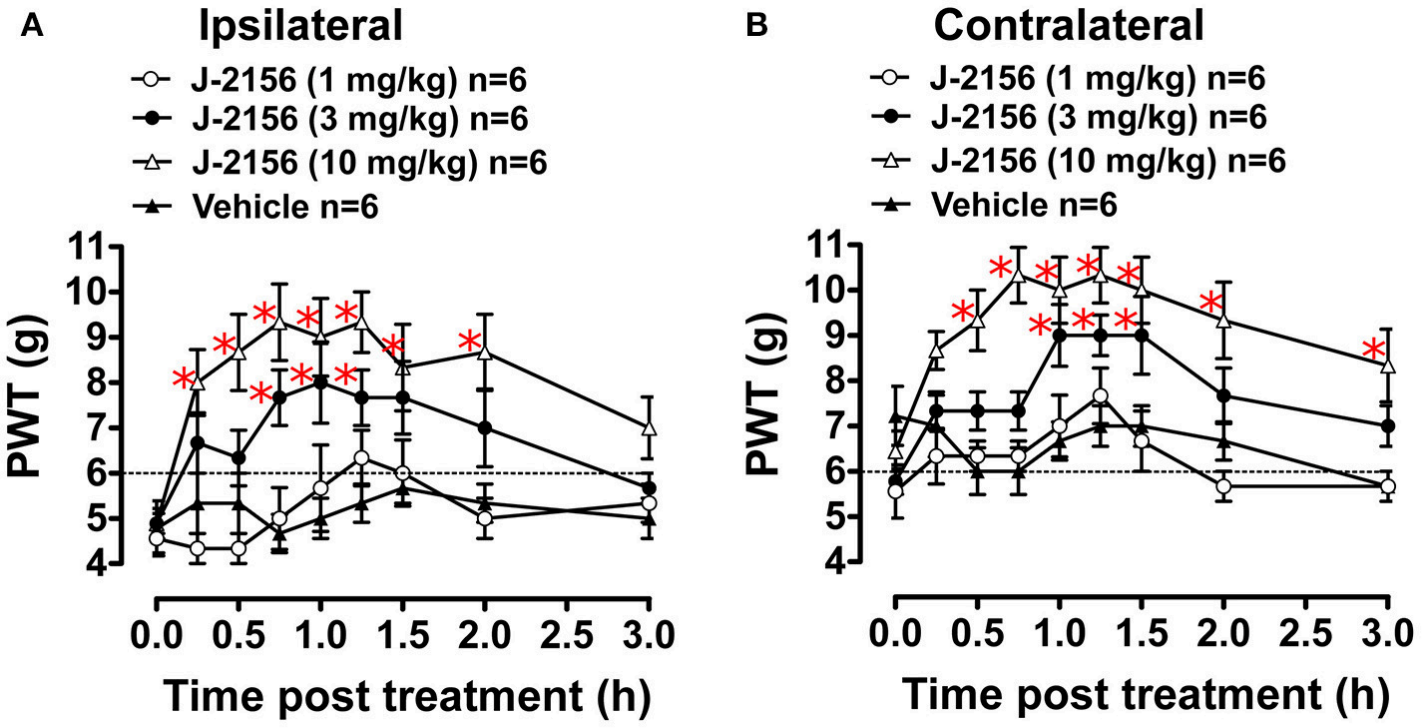
Anti-allodynic effect of single bolus doses (i.p.) of J-2156 on ipsilateral and contralateral hindpaw withdrawal thresholds (PWTs) in BCIBP-rats. Panels in the figure show **(A)** ipsilateral PWT vs. time curves and **(B)** contralateral PWT vs. time curves. The dotted line shows the threshold criterion of fully developed mechanical allodynia (≤6 g). Red asterisks ^*^p ≤ 0.05 [ipsilateral: *F*_(3, 8, 24/160)_ = 20.5, 6.4, 1.5; contralateral: *F*_(3, 8, 24/160)_ = 21.6, 8.0, 1.8; Two-way ANOVA, *post-hoc* Bonferroni test *c.f*. BCIBP-rats administered vehicle]. Between-dose differences (*p* ≤ 0.05) in the PWT values were also present.

Administration of J-2156 to BCIBP-rats produced dose dependent anti-allodynia in both the ipsilateral and the contralateral hindpaws (Figure 3). The ED_50−Ipsilateral_ and ED_50−Contralateral_ of J-2156 against mechanical allodynia in the BCIBP-rats were found to be 3.7 mg/kg (95% confidence interval, 2.5–5.4) and 6.6 mg/kg (95% confidence interval, 4.2–10.5), respectively.

### Anti-hyperalgesic effect of J-2156 in BCIBP

**Figure 4:**
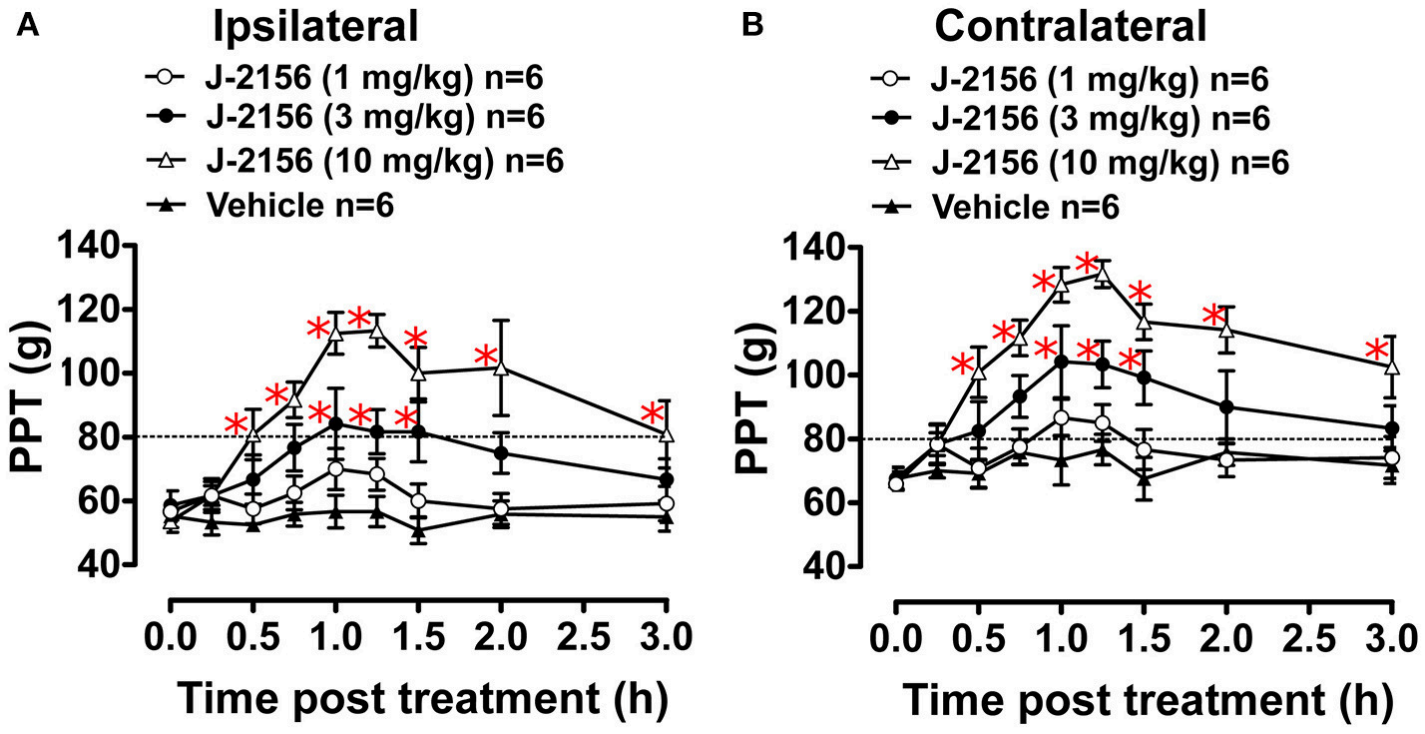
Anti-hyperalgesic effect of single bolus doses (i.p.) of J-2156 on ipsilateral and contralateral hindpaw pressure thresholds (PPTs) in BCIBP rats. Panels in the figure show **(A)** ipsilateral PPT vs. time curves and **(B)** contralateral PPT vs. time curves. The dotted line shows the threshold criterion of fully developed mechanical hyperalgesia (≤80 g). Red asterisks ^*^p ≤ 0.05 [ipsilateral: *F*_(3, 8, 24/160)_ = 15.0, 10.1, 3.1; contralateral: *F*_(3, 8, 24/160)_ = 19.3, 13.2, 2.5; Two-way ANOVA, *post-hoc* Bonferroni test *c.f*. BCIBP-rats administered vehicle]. Between-dose differences (*p* ≤ 0.05) in the PPT values were also present.

Administration of J-2156 to BCIBP-rats produced dose dependent anti-hyperalgesia in both the ipsilateral and the contralateral hindpaws (Figure 4). The ED_50−Ipsilateral_ and ED_50−Contralateral_ of J-2156 for relief of mechanical hyperalgesia in the BCIBP-rats were found to be 8.0 mg/kg (95% confidence interval, 5.3–12.2) and 5.0 mg/kg (95% confidence interval, 3.6–6.8), respectively.

### Validation of the anti-SST4 receptor antibody

In coronal sections of rat brain used as a positive control, the SST4 receptor antibody produced immunofluorescence consistent with expectations. Importantly, in sections of rat liver that was used as a negative control, SST4 receptor immunofluorescence was absent (Supplementary Figure 1). The immunofluorescence patterns of the anti-SST4 receptor antibody using the afore-mentioned positive and negative control sections demonstrated its specificity for the SST4 receptor and absence of cross-reactivity with other somatostatin receptors, thereby validating the antibody.

### Expression of SST4 receptor in DRGs and spinal cord in BCIBP

**Figure 5:**
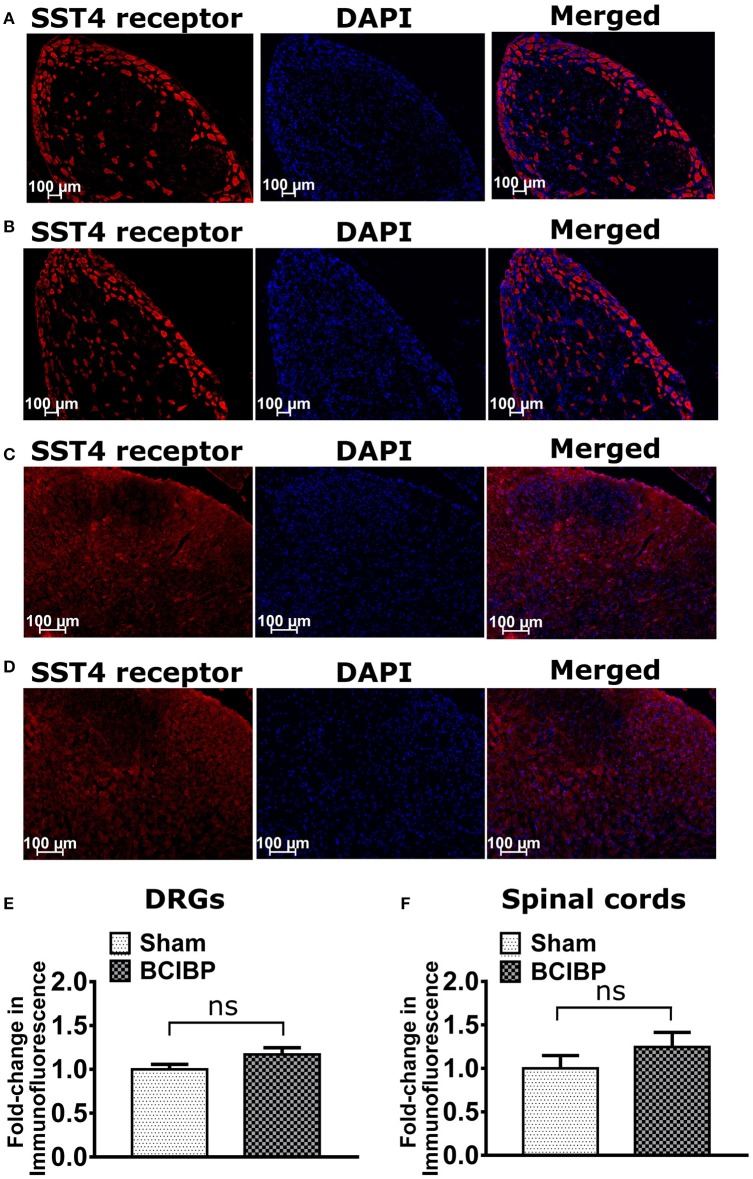
Expression levels of SST4 receptor in sections of ipsilateral lumbar L4-L6 dorsal root ganglia (DRGs) and lumbar spinal dorsal horns of BCIBP-rats and the corresponding sections from sham rats (*n* = 3–4/group). Panels in the figure show representative sections of **(A)** DRG of a sham rat, **(B)** DRG of a BCIBP-rat, **(C)** spinal dorsal horn of a sham rat and **(D)** spinal dorsal horn of a BCIBP-rat. Panel **(E)** shows fold-change in immunofluorescence of DRG sections of the BCIBP group relative to the sham group and **(F)** shows fold-change in immunofluorescence of lumbar spinal cord sections of the BCIBP group relative to the sham group. ns, statistically not significant (*p* > 0.05, Mann-Whitney test).

Expression levels of the SST4 receptor in sections of either the lumbar DRGs or the lumbar spinal dorsal horns of BCIBP-rats did not change significantly (*p* > 0.05) *c.f*. the corresponding sections from sham rats (Figure 5).

### Expression of somatostatin in DRGs and spinal cord in BCIBP

**Figure 6:**
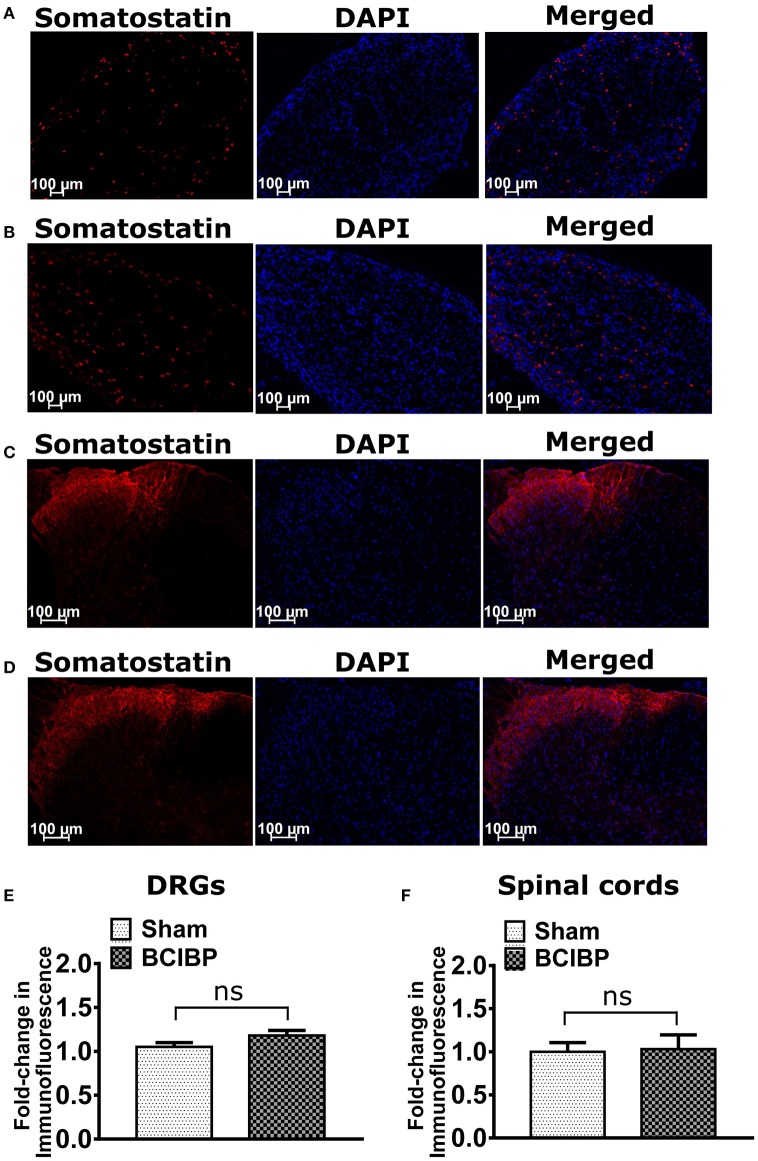
Expression levels of somatostatin in sections of ipsilateral lumbar L4-L6 dorsal root ganglia (DRGs) and spinal dorsal horns of BCIBP-rats and the corresponding sections from sham rats (*n* = 3–4/group). Panels in the figure show representative sections of **(A)** a lumbar DRG from a sham rat, **(B)** a lumbar DRG from a BCIBP-rat, **(C)** a lumbar spinal dorsal horn from a sham rat and **(D)** a lumbar spinal dorsal horn from a BCIBP-rat. **(E)** shows fold-change in immunofluorescence of DRG sections from the BCIBP group relative to the corresponding sections from the sham group and **(F)** shows the fold-change in immunofluorescence of lumbar spinal cord sections from the BCIBP group relative to the sham group. ns, statistically not significant (*p* > 0.05, Mann-Whitney test).

Expression levels of somatostatin in sections of either the lumbar DRGs or the lumbar spinal dorsal horns of BCIBP-rats did not change significantly (*p* > 0.05) *c.f*. the corresponding sections from sham rats (Figure 6).

### Distribution of the SST4 receptor in the ipsilateral lumbar DRGs of BCIBP-rats

**Figure 7:**
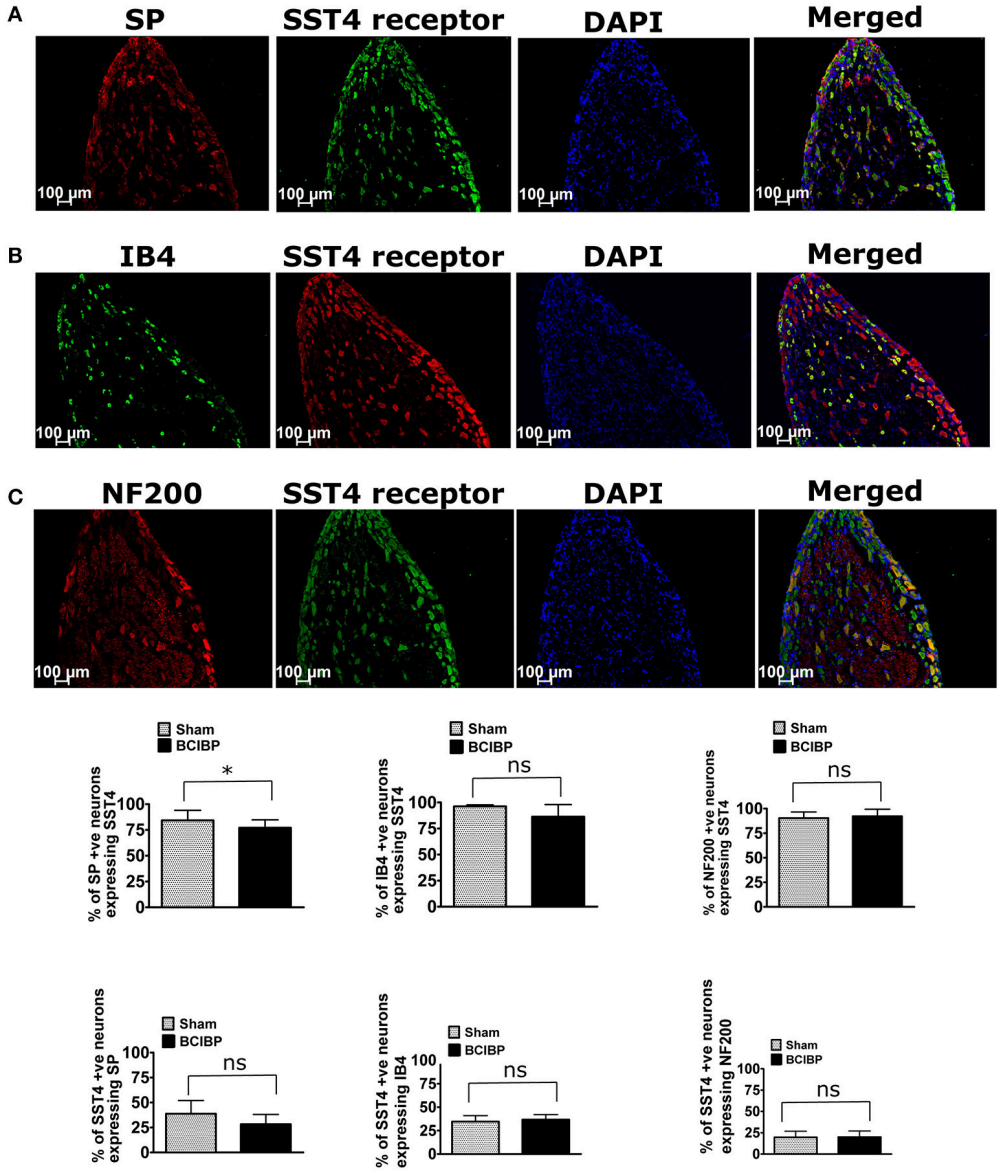
Immunostaining showing co-localization of the SST4 receptor with **(A)** substance P (SP), **(B)** isolectin B4 (IB4), and **(C)** neurofilament 200 kDa (NF200) in representative sections from ipsilateral lumbar L4-L6 dorsal root ganglia (DRGs) of BCIBP-rats (*n* = 3–4/group). ns, statistically not significant (*p* > 0.05); ^*^p ≤ 0.05 (Mann-Whitney test).

In BCIBP-rats, 77% of SP-positive neurons, 86% of IB4-positive neurons and 92% of NF200-positive neurons expressed the SST4 receptor. By comparison, from total SST4 receptor-positive neurons, 28, 37, and 20% were positive for SP, IB4, and NF200, respectively (Figure 7). This distribution profile in BCIBP-rats did not differ significantly (*p* > 0.05) from that of the sham-rats. Although, there was a statistically significant (*p* ≤ 0.05) decrease in the percentage of SP-positive neurons expressing the SST4 receptor in BCIBP-rats *c.f*. sham rats, this change was only marginal (~5%) and probably not physiologically relevant.

### Effect of J-2156 on pERK levels of spinal cord in BCIBP

**Figure 8:**
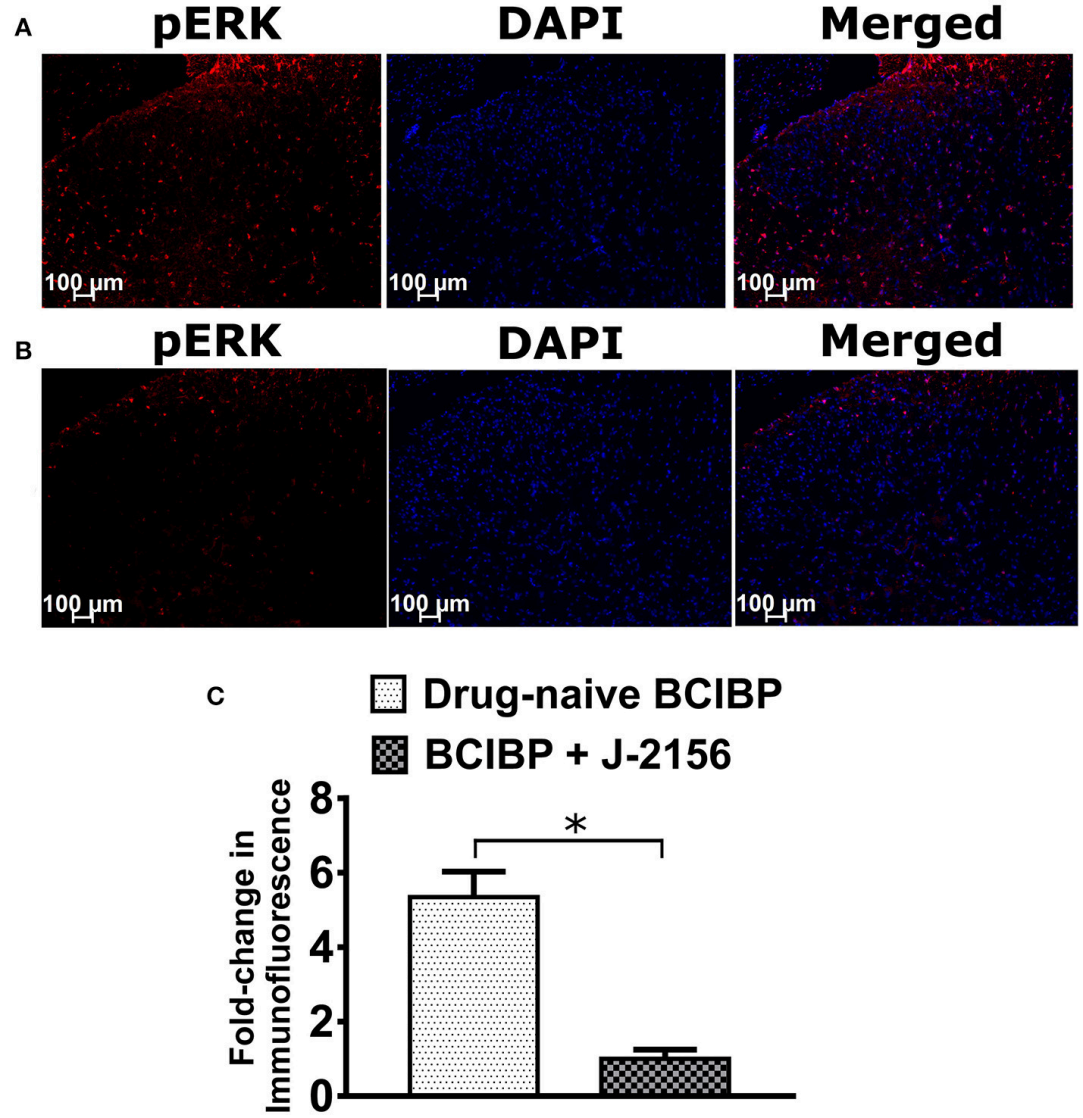
Effect of a single bolus dose of J-2156 (10 mg/kg, i.p.) on expression levels of phosphorylated extracellular signal-regulated kinase (pERK) in lumbar L4-L6 spinal dorsal horns of BCIBP-rats (*n* = 3–4/group). Panels in the figure show **(A)** representative section from a drug-naïve BCIBP-rat, **(B)** representative section from a BCIBP-rat administered J-2156 (10 mg/kg, i.p.) and **(C)** fold-change in immunofluorescence of sections from the BCIBP group administered J-2156 relative to the corresponding sections from the drug-naïve BCIBP group. ^*^p ≤ 0.05 (Mann-Whitney test).

Several previous studies have established that pERK expression levels are elevated in the lumbar spinal cord of rats receiving a unilateral ITI of Walker 256 cells, in the pain state (Wang L.-n. et al., 2011; Chen et al., 2012; Hu et al., 2015; Zhu et al., 2015; Ding et al., 2017; Sun et al., 2017). In BCIBP-rats administered a bolus dose of J-2156 at 10 mg/kg i.p., pERK expression levels in the lumbar spinal dorsal horns at the time of peak effect of anti-hypersensitivity were significantly (*p* ≤ 0.05) decreased *c.f*. the corresponding sections from drug-naïve BCIBP-rats (Figure 8).

## Discussion

We are the first to show that the small molecule SST4 receptor agonist, J-2156 (Prévôt et al., 2017), evokes dose-dependent relief of both mechanical allodynia and mechanical hyperalgesia in the bilateral hindpaws in a rat model of BCIBP. For the ipsilateral hindpaws, the ED_50_ values for J-2156 induced anti-allodynia and anti-hyperalgesia were 3.7 mg/kg and 8.0 mg/kg, respectively. Mechanisms of pathophysiology underlying the allodynic and hyperalgesic states could be diverse (Jensen and Finnerup, 2014), and hence the potencies of drugs in these states can differ significantly. Compared to allodynia, the higher ipsilateral ED_50_ value of J-2156 against hyperalgesia, is in agreement with higher anti-hyperalgesic ED_50_ values typically observed with other analgesic drugs (Espinosa-Juárez et al., 2017). We could not assess the effect of J-2156 on thermal nociceptive thresholds, as our previous experiments revealed that thermal hyperalgesia is not developed in this model (Shenoy et al., 2017). Consistent with these pharmacology data, the SST4 receptor was expressed by the majority of peripheral somatosensory neurons in the ipsilateral lumbar DRGs in BCIBP-rats. The distribution pattern of the SST4 receptor is consistent with its proposed role in modulating pain and transducing endogenous pain relief. Additionally, our findings herein show, for the first time, that a single bolus dose of J-2156 at 10 mg/kg reduced pERK expression levels in the lumbar spinal dorsal horn of BCIBP-rats.

Amongst published studies on the use of the Walker 256 cell induced BCIBP model, some have reported the development of unilateral (ipsilateral) hindpaw hypersensitivity (Liu et al., 2010; Tong W. et al., 2010), while others observed bilateral (ipsilateral and contralateral) hindpaw hypersensitivities (Mao-Ying et al., 2006, 2012). We have shown in our recently published study that pain hypersensitivities in this model are unilateral following intratibial injection of a low number of Walker 256 cells, with bilateral hindpaw hypersensitivities becoming evident following injection of a larger number of cells (Shenoy et al., 2017). With the number of Walker 256 cells inoculated in the tibiae of rats in the present study, we observed bilateral hindpaw hypersensitivities. Peripheral mechanisms like circulating factors and transmedian sprouting as well as centrally acting mechanisms like involvement of signaling via commissural interneurons of the spinal cord might underpin contralateral mirror effects associated with unilateral injury (Koltzenburg et al., 1999). Breast cancer cells in the bone cause sprouting of sensory nerve fibers innervating the periosteum and these persistent peripheral noxious inputs can sensitize certain parts of the brain to trigger bilateral hypersensitivities via modulation of descending pain control signaling (Ikeda et al., 2007; Meeus and Nijs, 2007). Activation of spinal glial cells and secretion of proinflammatory cytokines can also be responsible for the contralateral effects (Chacur et al., 2001).

The SST4 receptor is present in multiple tissues including brain, pancreas, stomach, lungs, placenta and kidney (Caron et al., 1997; Selmer et al., 2000a,b; Weckbecker et al., 2003; Bhandari et al., 2008). Additionally, SST4 receptor mRNA is widely distributed in both the periphery and the central nervous system (Bruno et al., 1993; Fehlmann et al., 2000; Ludvigsen et al., 2015). In female Lewis rats at ~10 weeks of age, SST4 receptor immunoreactivity was present in about 40% of DRG neurons as well as in some satellite cells of the peripheral nervous system (Bär et al., 2004). SST4 receptor immunoreactivity has been shown to be present in both the dorsal and ventral horns of the rat spinal cord (Somvanshi and Kumar, 2014), as well as in astrocytes (Feindt et al., 1995), and microglia (Feindt et al., 1998). Our findings of expression of the SST4 receptor in lumbar spinal cord and lumbar DRGs of rats are aligned with work by others, showing the presence of the SST4 receptor in both the central and peripheral nervous system. These findings herein are in agreement with previous work by others, showing that the SST4 receptor mediates pain relief in rodent models of both inflammatory and neuropathic pain (Helyes et al., 2006, 2009; Sándor et al., 2006; Elekes et al., 2008; Varecza et al., 2009; Szolcsányi et al., 2011; Schuelert et al., 2015). In other work, the δ-opioid receptor was heterodimerized with the SST4 receptor in the physiological state, thereby raising the possibility of potentiated endogenous pain relief (Somvanshi and Kumar, 2014). However, whether such a possibility of receptor heterodimerization exists in the pathophysiological state of cancer induced bone pain, remains to be investigated. In support of this notion, SST4 receptor gene knockout mice exhibited exaggerated inflammation and hyperalgesia compared with their wild-type counterparts (Helyes et al., 2009). Using gene knockout mice, the SST4 receptor was shown to be coupled to the K^+^ M-current (Qiu et al., 2008), which has the potential to modulate pain behaviors (Tsantoulas and McMahon, 2014). All somatostatin receptors, including the SST4 receptor, signal via inhibition of the adenylyl cyclase-cAMP pathway (Bruns et al., 1995). This pathway is the best characterized effector system associated with opioid receptor signaling (Law et al., 2000; Lantero et al., 2014) and its role in pain pathobiology and analgesia is very well established (Pierre et al., 2009; Sadana and Dessauer, 2009).

Administration of single bolus doses of J-2156 (i.p.) alleviated Complete Freund's adjuvant (CFA) induced- inflammatory pain at 0.1–1.0 mg/kg in male Han-Wistar rats (Schuelert et al., 2015) and at 0.001–0.01 mg/kg in male Lewis rats (Sándor et al., 2006). Similarly, J-2156 at 0.1 mg/kg alleviated carrageenan-induced inflammatory pain (Helyes et al., 2009) and at 0.01–0.1 mg/kg (i.p.) alleviated formalin-induced inflammatory pain in mice (Sándor et al., 2006). Additionally, J-2156 at 0.01–0.1 mg/kg (i.p.) alleviated neuropathic pain behaviors in rats with sciatic nerve ligation (Sándor et al., 2006). However, we required higher doses of J-2156 to observe alleviation of BCIBP in this model. This could perhaps be due to the fact that, unlike other pain models, cancer-associated pain pathophysiology concurrently involves inflammatory, neuropathic and tumor-specific components. Assessing whether the anti-hypersensitivity effects of J-2156 are ablated by pre-treating the animals with an SST4 receptor antagonist, would have been an interesting additional experiment to our work. However, fully characterized SST4 receptor selective antagonists are not yet available (Helyes et al., 2009). Additionally, the Walker 256 breast cancer cell induced bone pain model is syngeneic to rat species (Shenoy et al., 2016), hence we also could not use SST4 gene knockout mice to assess the ablation of analgesic effect produced by J-2156 in this model. Nevertheless, our *in vitro* off-target pharmacology assessment data, and affinity and potency data of J-2156 showed no significant binding and affinity of J-2156 to other non-cognate targets including the somatostatin receptor types 1, 2, 3, and 5.

In the present study in BCIBP-rats, there were no changes in the expression levels of either the SST4 receptor or its ligand, somatostatin, in neural tissue sections analyzed. Our findings are in agreement with those of a previous study whereby neither expression levels of the SST4 receptor nor the proportion of neurons expressing the SST4 receptor changed in the lumbar DRGs of rats with unilateral antigen-induced arthritis in the knee joint (Bär et al., 2004).

Sensory nerve fibers are in close proximity with cancer cells colonizing the bones, in a micro-environment that is suitable for activation and sensitization of these primary afferents (di Mola and di Sebastiano, 2008; Sroka et al., 2010; Voss and Entschladen, 2010; Mancino et al., 2011; Cole et al., 2015; Jobling et al., 2015; Yoneda et al., 2015). Functional interactions with cancer cells result in hyperexcitability and changes to the morphology of peripheral nerves (Cain et al., 2001), thereby causing pain hypersensitivities (Sughrue et al., 2008). Tumor tissue and immune cell derived endogenous substances such as nerve growth factor have the potential to cause sprouting of primary sensory nerve fibers with the net effect being bone pain (Tong Z. et al., 2010). Specifically, Walker 256 cells also secrete pro-inflammatory mediators (Rebeca et al., 2008; Pavlaki et al., 2009). Furthermore, cancer invasion and bone loss causes destruction of nerve endings of sensory neurons innervating the bones and the bone marrow, thereby causing intense hypersensitivities (Kane et al., 2015). Following intravenous administration of J-2156 at 5 mg/kg in rats with CFA- induced inflammatory pain, there was significant inhibition of primary afferent nerve firing, however, J-2156 showed no effect in the corresponding group of sham rats (Gorham et al., 2014b; Schuelert et al., 2015). Thus, J-2156 does not affect neuronal transmission under normal physiological conditions (Gorham et al., 2014b; Schuelert et al., 2015). While the endogenous ligand, somatostatin is well-known for its inhibitory actions on primary afferent nerve fibers (Carlton et al., 2004; Guo et al., 2008; Wang et al., 2009; Luo et al., 2010; Wang J. et al., 2011), the peptidomimetic compound- J-2156 (Sándor et al., 2006) inhibits transient receptor potential vanilloid 1 (TRPV1) currents, activates G-protein coupled inwardly rectifying potassium channels (GIRK) and inhibits voltage stimulated calcium channels in rat DRG neurons by specifically acting on SST4 receptors (Gorham et al., 2014a,b). It remains to be investigated whether J-2156 can inhibit the primary afferent nerve firing in the pathophysiological state of BCIBP. However, as knowledge on the DRG neuronal subtypes that express SST4 receptors was lacking, we performed co-localization experiments with subtype specific markers to address this open question. Here we show for the first time that the SST4 receptor is expressed by the vast majority of small diameter peptidergic and non-peptidergic lumbar DRG neuronal cell bodies as well as cell bodies from medium/large diameter lumbar DRG neurons.

The adenylyl cyclase-cAMP pathway, coupled to the extracellular signal-regulated kinase pathway via a small G-protein, Rap1, acts upstream of ERK activation and contributes to the induction of pERK in spinal cord (Kawasaki et al., 2004; Wei et al., 2006). pERK is a prominent player in the development of neural plasticity, which is a key pathobiological event in the development and maintenance of chronic pain (Ji et al., 2003, 2009; Stamboulian et al., 2010; Andres et al., 2013). pERK is only induced by noxious stimuli and not by normally innocuous stimuli and its inhibition is generally utilized in the assessment of analgesic efficacy and pain relief mechanisms of novel analgesic compounds (Ji et al., 2009). pERK is very dynamic in nature as the levels of this protein can elevate as well as deplete within a few minutes (Ji et al., 1999; Gao and Ji, 2009; Muralidharan et al., 2014). pERK levels in the spinal dorsal horns are instantly modulated following noxious peripheral stimuli (Ji et al., 1999). After intraperitoneal administration of J-2156 at 1 mg/kg to adult male Han-Wistar rats, the cerebrospinal fluid (CSF) concentration of J-2156 was below the detection limit, thereby indicating that this compound is unlikely to penetrate the blood-brain barrier to a significant extent, consistent with its lack of discernible CNS side-effects in the present study (Schuelert et al., 2015). Both somatostatin and SST4 receptor-specific agonism can reduce pERK levels (Hubina et al., 2006; Somvanshi and Kumar, 2014). We are the first to show that administration of a SST4 receptor specific agonist J-2156, reduces the pERK levels in the spinal dorsal horn of BCIBP-rats. In this context, it is plausible that J-2156 decreases primary afferent hyper-excitability, thereby indirectly reducing expression levels of pERK in the lumbar spinal dorsal horn, despite J-2156 not having crossed the blood-brain barrier. However, this remains for future investigation.

The doses of J-2156 that produced pain relief were higher in BCIBP-rats than those reported previously in other rodent pain models. Importantly however, there were no discernible side effects observed in any BCIBP-rat dosed with J-2156. No signs of sedation were observed in the animals at any of the doses of J-2156 tested herein. This good tolerability of J-2156 is consistent with the findings in humans showing that intravenous infusion of somatostatin in patients with abdominal pain associated with pancreatitis, was well-tolerated (Concepción-Martín et al., 2014). Similarly, administration of TT232, a somatostatin analog acting through peripheral SST4 receptors was devoid of significant toxicity or side effects in humans (Szolcsányi et al., 2004; Szokolóczi et al., 2005). Octreotide can alleviate pain behavior in patients (Penn et al., 1992; Dahaba et al., 2009). However, given its *in vitro* selectivity profile, octreotide's analgesic effect is most likely independent of SST4 receptor and more related to activation of SST2 receptor (Imhof et al., 2011). In alignment to this fact, the anti-inflammatory and the anti-hyperalgesic effects of octreotide in antigen induced arthritic mice were mediated via the SST2 receptor as these effects were abolished in SST2 knockout mice (Imhof et al., 2011). SST4 receptor mediated alleviation of pain behaviors induced by J-2156 in the present study is consistent with a pharmacological study by others showing that the pain-relieving effects of J-2156 are abolished in mice null for the SST4 receptor (Helyes et al., 2009). The findings presented herein, for the first time, show significant potential of the SST4 receptor in alleviating the complex symptomatology of BCIBP.

## Author contributions

All authors listed have made a substantial, direct and intellectual contribution to the work, and approved it for publication.

### Conflict of interest statement

The authors declare that the research was conducted in the absence of any commercial or financial relationships that could be construed as a potential conflict of interest.

## Abbreviations

AUC: area under curve
BCIBP: breast cancer induced bone pain
ITI: intra-tibial injection
PPT: paw pressure threshold.
