# Supplementary material for: The Somatostatin Receptor-4 Agonist J-2156 Alleviates Mechanical Hypersensitivity in a Rat Model of Breast Cancer Induced Bone Pain
Source: Front Pharmacol. 2018 May 15;9:495. doi: 10.3389/fphar.2018.00495 (PMC5962878; doi:10.3389/fphar.2018.00495)
Supplement: Supplementary file 1 [file Table_1.pdf]

Supplementary Material

The Somatostatin Receptor-4 Agonist J-2156 Alleviates Mechanical Hypersensitivity in a Rat Model of Breast Cancer Induced Bone Pain

Priyank Shenoy[1], Andy Kuo[1], Nemat Khan[1], Louise Gorham[2], Janet R. Nicholson[2], Laura Corradini[2], Irina Vetter[3,4], Maree T. Smith[1,4]*

[1] School of Biomedical Sciences, Faculty of Medicine, The University of Queensland, Brisbane, QLD, Australia

[2] Department of CNS Diseases Research, Boehringer Ingelheim Pharma GmbH & Co. KG, Biberach, BW, Germany

[3] Institute for Molecular Bioscience, The University of Queensland, Brisbane, QLD, Australia

[4] School of Pharmacy, Faculty of Health and Behavioural Sciences, The University of Queensland, Brisbane, QLD, Australia

***Corresponding Author:**
Professor Maree T. Smith
Email: maree.smith@uq.edu.au

Address:
Director, Centre for Integrated Preclinical Drug Development (CIPDD),
School of Biomedical Sciences
Faculty of Medicine
The University of Queensland
Brisbane, QLD 4072
Australia.
Institutional URL: https://cipdd.centre.uq.edu.au/home

Tel: +61-7-33652554
Fax: +61-7-33467391

Supplementary Table 1. Binding of J-2156 to various non-cognate pharmacological targets.

**Pharmacological Target**	**Species**	**Percent (%) inhibition of binding**
Adenosine A_1	human	19
Adenosine A_{2A}	human	-3
Adenosine A_3	human	-13
Adrenergic α_{1A}	rat	-11
Adrenergic α_{1B}	rat	8
Adrenergic α_{1D}	human	17
Adrenergic α_{2A}	human	4
Adrenergic β_1	human	0
Adrenergic β_2	human	6
Androgen (Testosterone) AR	rat	-2
Bradykinin B_1	human	23
Bradykinin B_2	human	-6
Calcium Channel L-Type, Benzothiazepine	rat	9
Calcium Channel L-Type, Dihydropyridine	rat	12
Calcium Channel N-Type	rat	-19
Cannabinoid CB_1	human	-4
Dopamine D_1	human	3
Dopamine D_{25}	human	9
Dopamine D_3	human	10
Dopamine $D_{4.2}$	human	-4
Endothelin ET_A	human	10
Endothelin ET_B	human	-1
Epidermal Growth Factor (EGF)	human	5
$GABA_A$, Flunitrazepam, Central	rat	6
$GABA_A$, Muscimol, Central	rat	-1
$GABA_{B1A}$	human	4
Glucocorticoid	human	6
Glutamate, Kainate	rat	-22
Glutamate, NMDA, Agonism	rat	14
Glutamate, NMDA, Glycine	rat	0
Glutamate, NMDA, Phencyclidine	rat	2
Histamine H_1	human	-6
Histamine H_2	human	4
Histamine H_3	human	2
Imidazoline I_2, Central	rat	-5
Interleukin IL-1	mouse	-4
Leukotriene, Cysteinyl $CysLT_1$	human	-2
Melatonin MT_1	human	-1
Muscarinic M_1	human	-1
Muscarinic M_2	human	5
Muscarinic M_3	human	0
Neuropeptide Y Y_1	human	-1

Neuropeptide Y Y_2	human	-5
Nicotinic Acetylcholine	human	-2
Nicotinic Acetylcholine α1, Bungarotoxin	human	2
Opiate δ (OP1, DOP)	human	20
Opiate κ (OP2, KOP)	human	16
Opiate μ (OP3, MOP)	human	7
Phorbol Ester	mouse	-7
Platelet Activating Factor (PAF)	human	21
Potassium Channel [K_{ATP}]	hamster	0
Potassium Channel hERG	human	11
Prostanoid EP_4	human	-1
Purinergic P_{2X}	rabbit	5
Purinergic P_{2Y}	rat	-6
Rolipram	rat	9
Serotonin (5- Hydroxytryptamine) 5-HT_{1A}	human	24
Serotonin (5- Hydroxytryptamine) 5-HT_{2B}	human	1
Serotonin (5- Hydroxytryptamine) 5-HT_3	human	13
Sigma σ_1	human	-2
Sodium Channel, Site 2	rat	35
Tachykinin NK_1	human	25
Thyroid Hormone	rat	-4
Transporter, Dopamine (DAT)	human	6
Transporter, GABA	rat	6
Transporter, Norepinephrine (NET)	human	-19
Transporter, Serotonin (5-Hydroxytryptamine) (SERT)	human	-3

n=2; concentration=10 uM; +, inhibition of binding; -, enhanced binding

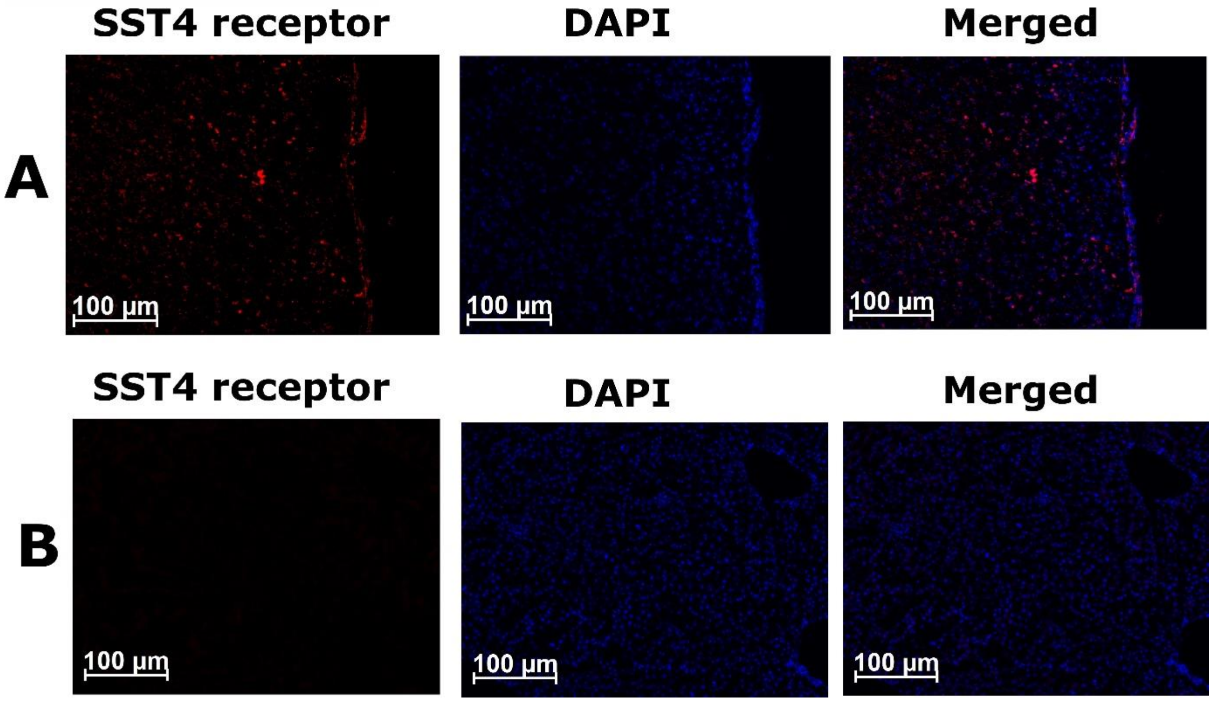

Supplementary Figure 1. Validation of the anti-SST4 receptor antibody using IHC staining. Panels in the figure show (A) a representative coronal section of rat brain used as a positive control for the SST4 receptor and (B) a representative rat liver section used as a negative control for the SST4 receptor.
